# Outcome of early active mobilization after flexor tendons repair in zones II–V in hand

**DOI:** 10.4103/0019-5413.65155

**Published:** 2010

**Authors:** Narender Saini, Vishal Kundnani, Purnima Patni, SP Gupta

**Affiliations:** Department of Orthopaedics, SMS Medical College, Jaipur, Rajasthan, India; 1 Bombay Hospital, Mumbai, SMS Medical College and attached group of Hospitals, Jaipur, Rajasthan, India; 2 Hand Surgery Unit, SMS Medical College and attached group of Hospitals, Jaipur, Rajasthan, India; 3 Orthopaedic Unit IV, SMS Medical College and attached group of Hospitals, Jaipur, Rajasthan, India

**Keywords:** Early mobilization, repair of flexor tendons, splints

## Abstract

**Background:**

The functional outcome of a flexor tendon injury after repair depends on multiple factors. The postoperative management of tendon injuries has paved a sea through many mobilization protocols. The improved understanding of splinting techniques has promoted the understanding and implication of these mobilization protocols. We conducted a study to observe and record the results of early active mobilization of repaired flexor tendons in zones II–V.

**Materials and Methods:**

25 cases with 75 digits involving 129 flexor tendons including 8 flexor pollicis longus (FPL) tendons in zones II–V of thumb were subjected to the early active mobilization protocol. Eighteen (72%) patients were below 30 years of age. Twenty-four cases (96%) sustained injury by sharp instrument either accidentally or by assault. Ring and little finger were involved in 50% instances. In all digits, either a primary repair (*n*=26) or a delayed primary repair (*n*=49) was done. The repair was done with the modified Kessler core suture technique with locking epitendinous sutures with a knot inside the repair site, using polypropylene 3-0/4-0 sutures. An end-to-end repair of the cut nerves was done under loupe magnification using a 6-0/8-0 polyamide suture. The rehabilitation program adopted was a modification of Kleinert’s regimen, and Silfverskiold regimen. The final assessment was done at 14 weeks post repair using the Louisville system of Lister *et al*.

**Results:**

Eighteen of excellent results were attributed to ring and little fingers where there was a flexion lag of < 1 cm and an extension lag of < 15°. FPL showed 75% (n=6) excellent flexion. 63% (*n*=47) digits showed excellent results whereas good results were seen in 19% (*n*=14) digits. Nine percent (*n*=7) digits showed fair and the same number showed poor results. The cases where the median (n=4) or ulnar nerve (*n*=6) or both (n=3) were involved led to some deformity (clawing/ape thumb) at 6 months postoperatively. The cases with digital or common digital nerve involvement (*n*=7 with 17 digits) showed five excellent, two good, four fair, and six poor results. Complications included tendon ruptures in 2 (3%) cases (one thumb and one ring finger) and contracture in 2 (3%) cases whereas superficial infection and flap necrosis was seen in 1 case each.

**Conclusion:**

The early active mobilization of cut flexor tendons in zones II–V using the modified mobilization protocol has given good results, with minimal complications.

## INTRODUCTION

The restoration of digital function after a flexor tendon injury continues to be the greatest challenge in the field of hand surgery. Scarring, adhesion formation, and subsequent stiffness have been the major hindrance to good results after a flexor tendon repair. The functional outcome of a flexor tendon injury after a repair depends on multiple factors such as age, injury level and type, type of repair, and post repair therapy. Most variables except mobilization protocols have been established and defined in the past.^1^–^5^

The postoperative management of tendon injuries has paved a sea through many mobilization protocols,^1^–^5^ each having its own merits and demerits. The ultimate aim of all postoperative rehabilitation protocols is the same –“Strong tendon that glides freely.” In last 100 years, the management of tendon injuries has not only seen advances in primary care, repair technique, suture technique, understanding of biomechanics and postoperative evaluation protocol,^6^–^13^ but also a drastic change in mobilization protocols ranging from strict immobilization to early/delayed active mobilization.

The improved understanding of splinting techniques has promoted these mobilization protocols. It has been proven that postoperative immobilization leads to increased disability period, weak tensile strength, decreased final functional capacity, stiffness, and deformity.^14^ Further early postoperative mobilization leads to improved tendon healing, increased tensile strength, decreased adhesion formation, early return of function, and less stiffness and deformity as compared to the immobilization protocol. However, as any other procedure it has its own demerits in the form of rupture of repaired tendons. We conducted a study to evaluate the outcome of early active mobilization of repaired flexor tendons in zones II–V.

## MATERIALS AND METHODS

34 patients with cut flexor tendons in zones II–V who reported during the study period of 2 years (from Nov 2004 to Oct 2006) constitute the clinical material. Out of these, nine patients were lost to follow-up and were thus excluded. 18 (72%) of our patients were below 30 years of age, with 52% (*n*=13) being in the 21-30 year age group. 23 of our patients were males. 24 sustained injury by sharp instrument either accidentally or by assault; one case was of suicidal attempt. Patients (*n*=25) with cut flexors in zones II–V with or without an associated vessel or nerve injury and presenting within 7 days of the injury were included in the study. Patients with fracture, simultaneous injury to extensor tendons, gross contamination of wound, and massive skin loss, psychologically impaired and noncompliant patients, and children less than 8 years of age were excluded from the study.

**Figure 1:**
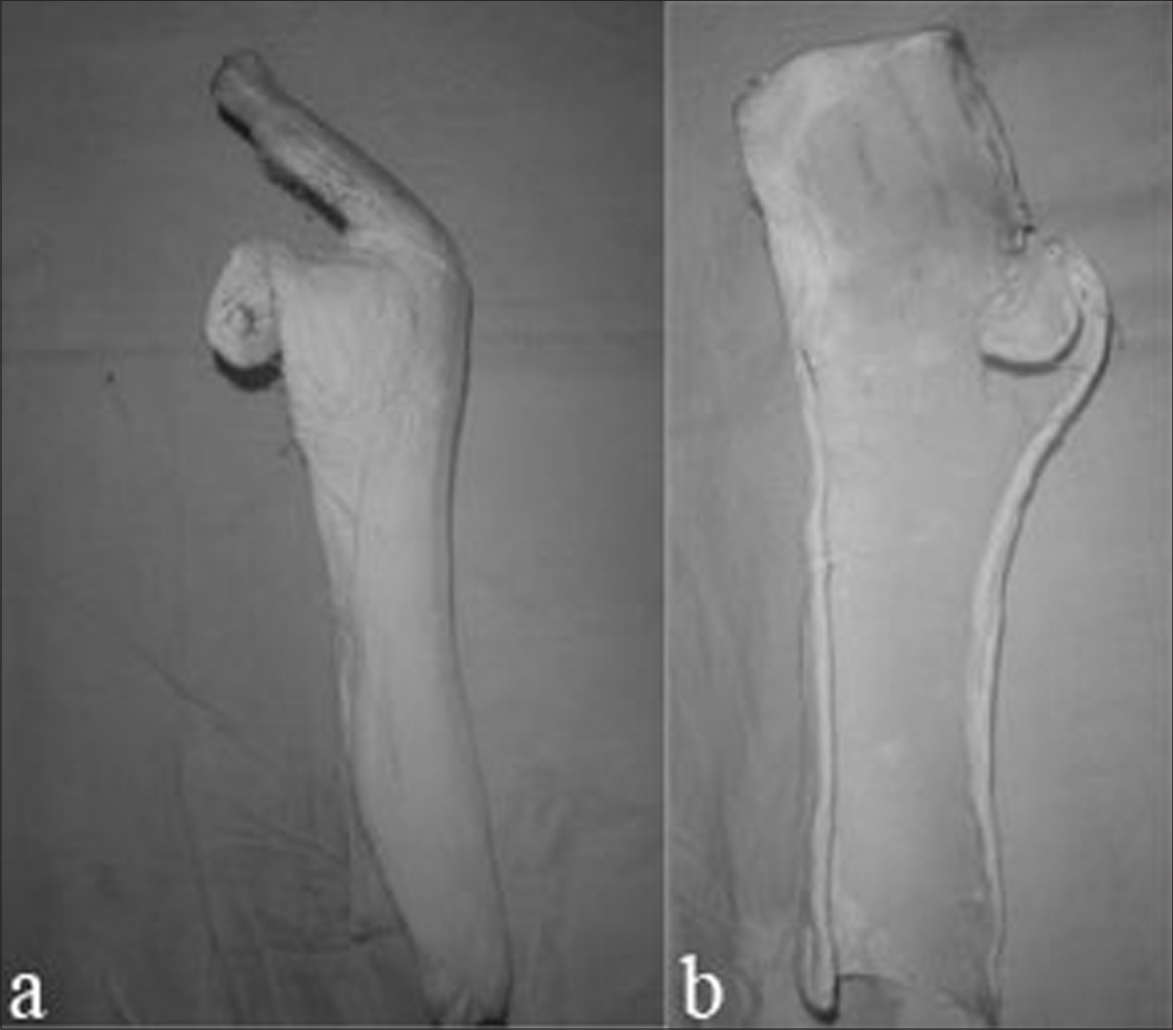
Photographs (a, b) showing the self made plaster of paris Splint

In all cases except one where secondary repair was done, either a primary repair (*n*=26 fingers in 9 patients) or a delayed primary repair (*n*=49 fingers in 15 patients) was done, under axillary block or general anesthesia with tourniquet control. The primary repair was done within 6–8 h of injury where the wound was clean, while in others either due to late presentation or potential infection delayed primary repair was done. The initial management in patients with delayed primary repair constituted of debridement of wound with antibiotic cover and to make sure before surgery that there is no infection. The wounds were extended or opened as necessary to retrieve retracted tendons. The zone II wounds were extended with a palmar zig-zag incision or the modified Brunner lateral incision strictly following the surgical principles of Verdan.^15^–^16^ The flexor sheath was opened enough to facilitate the repair, the pulleys were not excised, and the damaged pulleys were repaired with a polypropylene 6-0 suture. In zones III–V, lazy S or L incisions were used to expose the cut tendon. In all cases, an end-to-end repair of the cut tendons was done after freshening of cut ends. The repair was done with the modified Kessler^17^ core suture technique with locking epitendinous sutures with a knot inside the repair site. The repair was done with polypropylene 3-0/4-0 sutures. The end-to-end repair of the cut nerves was done under loupe magnification using a 6-0/8-0 polyamide suture. 20 patients had nerve injury; 4 median, 6 ulnar, 3 both and 7 digital nerves. Vascular repair was not done in any case. Postoperative immobilization was done with a splint in 10°–15° palmar flexion of the wrist and 70° flexion of metacarpophalangeal joints and interphalangeal joints in mild flexion [Figure 1a, b].

Rehabilitation was started 24 h postoperatively. The rehabilitation program adopted was a modification of Kleinert's regime^18^ and Silfverskiold regime.^19^ The rehabilitation protocol consisted of active extension, with initial active flexion and later passive flexion by Kleinert’s^18^ regimen and then further passive flexion. The wrist following nerve repair was kept in 5° palmar flexion and in cases of ulnar nerve cut, Metacarpophalangeal joint (MCP) was kept at 90° flexion. A detailed rehabilitation protocol is shown in Table 1. The patient was strictly advised not to passively stretch the repaired tendons, not to remove the splint unless instructed, avoid holding the hand in a dependent position, and not to increase the exercise session by self.

**Table 1:** Rehabilitation protocol

1 to 28 days	Kleinert’s regimen (elastic bands) applied to all fingers with the elastic band extended from the nail to the volar aspect of wrist. Splint: Dorsal splint with wrist 0°–5° dorsiflexion, MCP 70° flexion, and IP full extension (if nerve repair was done, the wrist was kept in 5° palmar flexion and in cases of an ulnar nerve cut, MCP was kept at 90° flexion)
	Exercises: Shoulder, elbow, supination/pronation promoted
	Hand: 10 times/session and 3 sessions/day
	Step 1: Active extension of all fingers after tension on Kleinert’s bands released, gaining full extension at IP and MCP joints blocked only by a splint
	Step 2: Active flexion of all fingers to possible flexion position without a forceful effort
	Step 3: Release tension on Kleinert’s bands to bring added passive flexion of fingers by rubber band tension
	Step 4: Passively flex the fingers at IP joints with the help of other hand
4–8 weeks	Kleinert’s bands removed
	Splint: Intermittant, volar splint with wrist 10°–15° palmar flexion, MCP 70° flexion and IP extension; removed during exercise; scar mobilization done
	Exercises: Shoulder, elbow, and wrist exercises continued
	Hand: 10 times/session and 3 sessions/day
	Active tunnel block exercises with isolated FDP/FDS. Block FDP of all fingers and isolated FDS function, and block FDS of all fingers and do isolated FDP contraction. Actively make fist, curling of all fingers into flexion; release and open actively extending to full extent
	If PIP joint contracture was present, passive stretching was started in the volar splint with cotton roll padding. Passive overflexion and extension with tender strokes were promoted to keep hand supple
8–12 weeks	Volar splint in 15°–25° dorsiflexion, MCP 50°–70° flexion IP full extension (used only as night splint)
	Scar mobilization continued
	Power grip allowed; ball exercises five times each session
	Resume light work, food, drinking, button knots, etc.
	Avoid heavy work
	Exercises: Aggressive shoulder, wrist radioulnar joint, and elbow exercises
	Hand: Ball exercises with a soft sponge 20 times per session and 4 times/day
12–14 weeks	No splintage
	Stop scar mobilization
	Power grip continued
	Resume to daily household work but avoid heavy work
	Exercise: Hand – continue same as above with an increased frequency of 50 times per session and 5 sessions per day

The flexion lag was measured as the pulp-to-palm distance in centimeters, where as the extension lag was measured as the amount of extension remaining in degree, comparing to normal digits. Since we have a rehabilitation protocol for 12 weeks, hence the final assessment was done at 14 weeks postrepair using the Louisville system of Lister *et al*.^18^ [Table 2].

**Table 2:** Louisville system

Excellent	Flexion lag < 1 cm/extension lag < 15°
Good	Flexion lag 1–1.5 cm/extension lag 15°–30°
Fair	Flexion lag 1.5–3 cm/extension lag 30°–50°
Poor	Flexion lag >3 cm/extension lag > 50°

## RESULTS

Of the 75 digits, majority (*n*=40) were zone V injuries while injury in zones II, III, and IV included 4, 17, and 14 fingers, respectively. The thumb was involved in 8 instances, while index finger, middle finger, ring finger and little finger were Involved in 14, 17, 22, 14 instances respectively. The study involved 8 FPL, 66 Flexor digitorum superficialis (FDS), and 55 Flexor digitorum profundus(FDP) tendons. A total of 96% (*n*=24) of our cases were repaired within 7 days and only one case was repaired after this period.

Thirteen cases where the median (*n*=4) or ulnar nerve (*n*=6) or both (*n*=3) were involved led to some deformity (clawing/ape thumb) at 6 months postoperatively. The ulnar nerve involvement was found to be more disabling due to clawing and intrinsic negative hand resulting in deficient MCP joint flexion and preventing IP joint extension hence decreasing the overall gliding and excursion of repaired tendons. The cases with digital or common digital nerve involvement (*n*=7 with 17 digits) showed five excellent, two good, four fair, and six poor results.

Isolated median nerve involvement did not pose much problem regarding the excursion of repair tendons. However, isolated ulnar nerve and both nerve injury hampered the excursion more commonly due to involvement of intrinsic muscles.

The average follow-up of each patient was 6 months. Results of tendon excursion were evaluated at 14 -16 weeks. The evaluation of functional ability/disability was done at the final follow-up (6 months).

Most (*n*=18) of the injuries due to accidents were in zones II, III, and V, whereas most of assault and suicidal injuries (*n*=7) involved zone V. A total of 64% (*n*=16) of our cases were injured in zones IV and V. Ring and little finger were most common digits to be involved (50%, *n*=13).

Three patients had single digit involvement, while the rest 22 patients had more than one digit involved. Twenty-one digits (including 8 thumbs) had single tendon involvement while 54 digits had more than one tendon involvement. All cases except one had either primary repair (26 digits and 9 patients) or delayed primary repair (48 digits and 15 patients).

Bulk of excellent results (*n*=26 fingers) were attributed by ring and little fingers where there was a flexion lag of < 1 cm and an extension lag of < 15°. FPL showed 75% (*n*=6 thumbs) excellent flexion and no poor results were seen in the middle finger. The poor results (five fingers) were mostly due to the flexion lag rather than extension lag [Tables 3 and 4]. Digits with a flexion lag did not necessarily show increased extension lag and thus a discrepancy existed in overall results as compared to the individual flexion/extension lag. However, if any of the above criteria showed poor/fair result, tendons were attributed to a lower grade.

**Table 3:** Flexion lag at the final evaluation of results

Digits	No. of digits	Up to 1 cm	1–2 cm	2–3 cm	>3 cm
Thumb	8	6	1	0	1
IF	14	7	5	1	1
MF	17	10	4	3	0
RF	22	15	4	2	1
LF	14	11	1	0	2

IF ‐ Index finger, MF ‐ Middle finger, RF ‐ Ring finger, LF ‐ Little finge

**Table 4:** Extension lag at final evaluation of results

Digits	No. of digits	<15°	16–30°	31–45°	>45°
Thumb	8	6	1	1	0
IF	14	9	5	0	0
MF	17	12	5	0	0
RF	22	17	5	0	0
LF	14	12	2	0	0

IF ‐ Index finger, MF ‐ Middle finger, RF ‐ Ring finger, LF ‐ Little finge

Due to discrepancy in the flexion and extension lag between each involved finger, tabulated results were evaluated/graded as per the Louisville criteria.^18^ Sixty-three percent (47/75) fingers showed excellent results whereas good results were seen in 19% (14/75) digits. Nine percent (7/75) digits showed fair and the same number showed poor results. On comparing the results zone-wise, it was observed that zone II results were poor in 25% cases and fair in equal number, whereas in zones III and IV, 60–70% excellent to good results were seen with the early active mobilization protocol. Excellent to good results were seen in 100% cases in zone V with this early mobilization protocol [Table 5]. [Figures 2A, 2B, 2C].

**Figure 2A:**
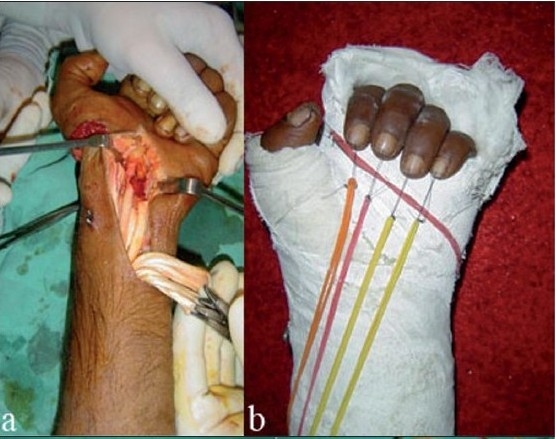
Clinical photographs of 31 yrs old male, assault with Axe showing (a) cut FPL, FDS/FDP of index, middle and ring fingers (ZONE IV), cut median nerve and radial artery with cut I and III extensor compartment and distal radius fracture. (b) Splint applied and mobilization taught.

**Figure 2B:**
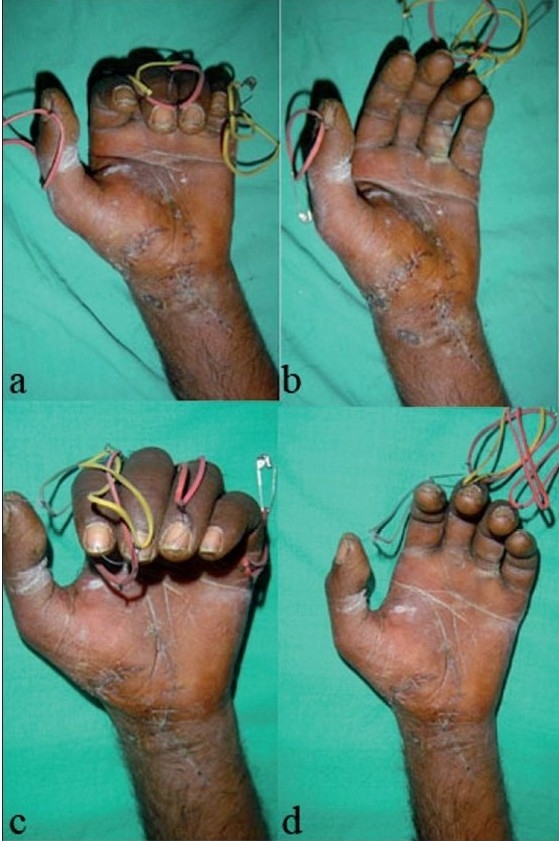
Clinical photographs showing (a) 2 weeks post operative- flexion of fingers. (b) 2 weeks post operative- extension of fingers. (c) 5 weeks post operative flexion of fingers. (d) 5 weeks post operative extension of fingers.

**Figure 2C:**
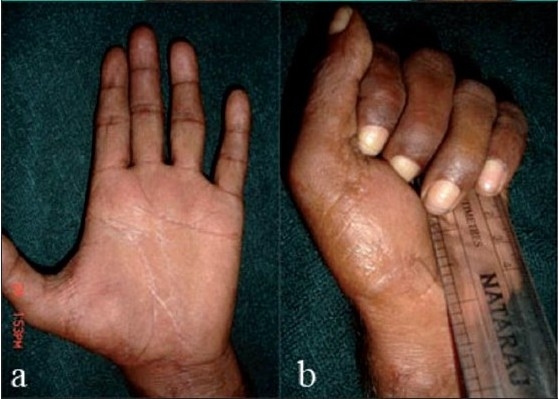
Clinical photographs at 8months follow up showing (a) extension with ape thumb. (b) flexion with 1 cm flexion lag.

Laceration occurring proximal to the carpal tunnel and involving wrist and finger flexors, median, ulnar or both nerves with both arteries cut also known as spaghetti wrist or full house syndrome was the most common pattern of injury in zone V. The involvement of ulnar structures with FCU, flexors of little and ring fingers with ulnar nerve and ulnar artery injury was the second most common pattern of involvement.

Five tendons in our study had frayed ends as compared to rest. The frayed tendons showed fair to poor results, whereas sharply cut tendons (*n*=124) showed excellent to good recovery.

Injuries occurring proximal to the carpal tunnel and involving wrist and finger flexors, and median or ulnar or both nerves with both arteries cut were the most common pattern of injury in zone V (12 patients). The involvement of ulnar structures with FCU, flexors of little and ring fingers with ulnar nerve, and ulnar artery injury was the second most common pattern of involvement [Figures 3A, 3B].

**Figure 3A:**
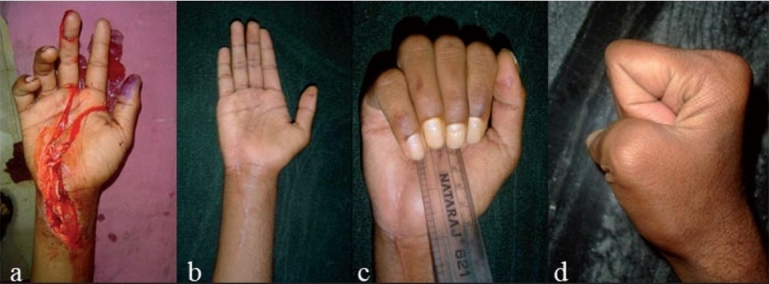
Clinical photographs 19 years old male cut right wrist due to assault (sword cut)showing (a) cut FPL, FDS/FDP of index and middle fingers, cut FDS of ring and little finger is Zone III, IV. cut median n, radial artery. (b), (c) 7 weeks post operative, showing good flexion. (d) At 24 weeks follow up- Right side showing good grip.

**Figure 3B:**
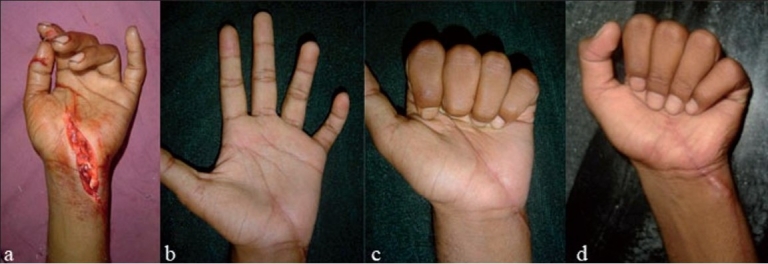
Clinical photographs of same patient with left side showing (a) Cut FDS/FDP ring and little finger in Zone IV, cut ulnar nerve, ulnar artery. (b), (c) 7 weeks post operative. (d) At 24 weeks follow up- showing good fist formation.

**Figure 4:**
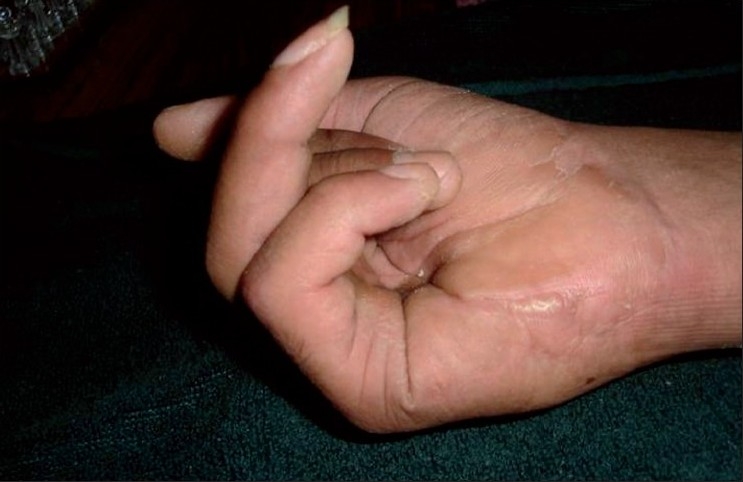
Clinical photographs showing complication- rupture of flexor tendon repair and Flexion lag

Some complications did occur. Ruptures were seen in 2 (3%) cases, in one FPL ruptured in zone VI and in another FDS/FDP of ring finger ruptured in zone II [Figure 4]. In both cases, there was a sudden loss of movement in the involved digit, and in both cases secondary repair with tendon graft was done. Contracture in 2 (3%) digits whereas superficial infection and flap necrosis was seen in 1 digit each. No tenolysis was required in our study. Then results of primary and delayed primary repair were identical.

**Table 5:** Zone-wise results

	Zone II (*n*=4)		Zone III (*n*=17)		Zone IV (*n*=14)		Zone V (*n*=40)	
	No.	%	No.	%	No.	%	No.	%
Excellent	2	50	6	35.29	6	42.85	35	87.5
Good	0	0	3	17.60	4	28.57	5	12.5
Fair	1	25	3	17.60	3	21.42		
Poor	1	25	5	29.41	1	7.14		

## DISCUSSION

Flexor tendon injuries are among the most common injuries of hand, occurring commonly in young males of the working class. Our study consisted of 25 patients, mostly males with a flexor tendon injury involving 75 digits with 129 tendons. We started our study to manage flexor tendon injuries with an aim to achieve full range of movement at IP, MCP, and wrist joints within 3.5 months with least possible complications.

Results after a flexor tendon injury repair are inversely proportional to the delay in the repair of the tendon^6^. The added benefits of a primary/delayed primary repair are decreased rehabilitation time, adhesion formation, and rupture rate, and increased healing rate with adequate tensile strength. Ninety-six percent of our cases were repaired within 7 days and only one case was repaired after this period.

Sharp cut tendons with transverse/oblique edges in our study showed better healing (*n*=124) and alignment as compared to those in which edges were found to be frayed. This has been earlier reported by Gault^11^ (1988) stating delayed vascularization in later cases to be the cause of poor healing.

Verdan *et al*.^15^ classified flexor tendon injury in digit and thumb in five zones and we have followed the same classification. A total of 53.5% (*n*=40) of digits in our study were injured in zone V, whereas 18.5% (*n*=14) in zone IV and 21% (*n*=17) in zone III. Zone V injuries commonly involved multiple tendons and had one/both nerves injured, whereas zone II and III injuries were more commonly reported to have a single tendon injury along with a neurovascular injury always associated.

Laceration occurring proximal to the carpal tunnel and involving wrist and finger flexors and median or ulnar or both nerves with both arteries cut also known as spaghetti wrist^21^ or full house syndrome was the most common pattern of injury in zone V. The involvement of ulnar structures with FCU, flexors of little and ring fingers with ulnar nerve, and ulnar artery injury was the second most common pattern of involvement.

Pucket and Meyer^21^ in there series of 38 spaghetti wrists achieved 87% good to excellent range of movement at IP, MCP, and wrist joints using active and passive mobilization protocol. Windgrow^22^ reported 19 cases using a definition of 10 injured structures including at least one major nerve and one major vessel called as spaghetti wrist. He reported good to excellent results in 95% of patients; however, protective sensation at 12 weeks was present in only 36% of cases, hampering the overall result. Hudson and Dejarger^23^ studied 15 patients with simultaneous laceration of the median and ulnar nerve of 76 repaired tendons. Their study showed 41% good to excellent results and 35% fair to poor results. In our study, 100% of tendon repair in zone V showed excellent to good results with protective sensation tested in the autonomous zone for each nerve present in 96% of cases at 14 weeks and average 2 PD (static two point discrimination) at 24 weeks to be at 9 mm. Our results are superior to any other study so far conducted.

Isolated median nerve involvement did not pose much problem regarding the excursion of repaired tendons. Although the involvement of the ulnar or median nerve did not affect the overall final gliding and excursion of tendons but excursion and gliding of tendon was more commonly hampered in early stages of spaghetti wrist. However, an isolated ulnar nerve injury hampered the excursion more commonly than any other form of nerve involvement and required some modification of splint (Flexion of MCP joints to 90° was done) to achieve good results. The overall return of function in all zones with nerve injuries was ultimately dependent on the return of protective sensation and final results were more hampered with median and ulnar nerve involvement as compared to the isolated injury to the nerve.

Pucket and Meyer^21^ and Windgrow^22^ showed that protective sensation was present in 40% of cases at 12 weeks and 2 PD at 24 weeks was 12 mm on average. The return of motor function was delayed up to 5 years in cases with median and ulnar injuries as reported by Rogers^24^ and only 48% of cases showed motor recovery at 2-year follow-up. In our series, 100% of cases involving median/ulnar or both showed ape thumb/clawing at 6-month follow-up and support the theory that motor recovery was late and never complete; however, protective sensation was present at average 12-14 weeks and return to activities of daily living was possible in all (96%) patients except one who had delayed return of protective sensation (24 weeks).In our study, we kept strict vigilance over cases with nerve injuries and modified our splints to overcome the factors hampering results and increase the excursion of tendons and their gliding properties^24^.

In ulnar nerve injury intrinsic negative hand lead to decrease of tendons excursion, due to clawing and hyperextension at MCP joints, attempting flexion was wasted at IP joints with a tendency to develop contracture at PIP joint and no flexion was brought at MCP joint, decreasing the overall gliding of the tendons. To overcome this we adjusted/changed the splint and kept the MCP dorsal block to 90° flexion and released Kleinert’s elastic rubber bands at 3 weeks.

Edinburg and Biddulph^25^ in their study of zone III injuries repaired primarily and active mobilization showed 71% fair to excellent and 29% poor results. In our study 42% of cases were in zones III and IV. 58% and 71% of results in zones III and IV, respectively, were reported with 29% excellent/good and 7% poor results, respectively.

In our study all tendons were repaired by modified Kessler’s core suture and continuous locking epitendinous sutures. This two-strand core suture not only gives adequate strength to the tendon but also prevents adding of bulk to the tendon which prevents gliding of the tendon in edematous repair zones and flexor sheath. Thurman^26^ compared strength between two-, four- and six-strand technique and stated that the two-/four-strand technique with modified Kessler/Tajima repair and epitendinous suture provides adequate strength to prevent rupture without adding bulk with an increased tensile strength of the repaired tendon. In all cases, a knot was placed inside the repair site as promoted by Aoki,^27^ Pruitt,^28^ and Mashadi.^29^

Early mobilization in our series showed a reduced rupture rate of 3% (two digits) as compared to 4-17% in other series.

The early active mobilization shows benefits of increased healing rate and tensile strength and decreased adhesion formation and rupture.^30^–^37^ The results are ranging from 70% excellent in Cullen^30^ and Chow^31^ to 100% excellent to fair in Silfverskiold.^34^ Our study showed 82% excellent to good results with nine fair and poor each as per the Louisville system criteria.^18^

In our series, eight FPL were repaired and subjected to early active mobilization. Eighty-seven percent (seven digits) showed excellent to good results and one had poor results due to the associated FPL rupture. Perceival and Sykes,^38^ Nunley,^39^ and Thomazean^40^ have used immobilization/controlled mobilization as the method of FPL rehabilitation with the rupture rate ranging from 8% to 17% and 44-71% showing excellent to good results. Our study has shown superior results in this context.

Complications reported in our series were skin flap necrosis in one case, managed surgically by a rotation flap, contracture at a PIP joint in two cases managed conservatively by Capner’s dynamic splinting, and rupture in two digits, 1 FPL zone V and 1 FDP zone II. The FPL rupture was cut and adherent with a slight functional deficit and did not require any surgery.

To conclude, primary or delayed primary repair of sharply cut flexor tendons with a modified Kessler core suture and locking epitendinous circumferential suture increases the overall strength, allowing active mobilization, which causes cyclic tension loading, leading to prevention of adhesions and good tendon healing. Thus the key to success for a flexor tendon repair lies in primary or delayed primary repair with early active mobilization protocol in a compliant patient having a zeal to get well soon.
